# Effects of Musical Training and Hearing Loss on Fundamental Frequency Discrimination and Temporal Fine Structure Processing: Psychophysics and Modeling

**DOI:** 10.1007/s10162-018-00710-2

**Published:** 2019-01-28

**Authors:** Federica Bianchi, Laurel H. Carney, Torsten Dau, Sébastien Santurette

**Affiliations:** 1 Hearing Systems Group, Department of Electrical Engineering, Technical University of Denmark, Ørsteds Plads, Building 352, 2800 Lyngby, Denmark; 2 Current Affiliation: Oticon Medical, Kongebakken 9, Smørum, Denmark; 3 Departments of Biomedical Engineering and Neuroscience, University of Rochester, Rochester, NY USA; 4 Department of Otorhinolaryngology, Head and Neck Surgery & Audiology, Rigshospitalet, 2100 Copenhagen, Denmark

**Keywords:** pitch discrimination, temporal fine structure, sensorineural hearing loss, musical training, Schroeder phase, auditory model

## Abstract

Several studies have shown that musical training leads to improved fundamental frequency (*F*_0_) discrimination for young listeners with normal hearing (NH). It is unclear whether a comparable effect of musical training occurs for listeners whose sensory encoding of *F*_0_ is degraded. To address this question, the effect of musical training was investigated for three groups of listeners (young NH, older NH, and older listeners with hearing impairment, HI). In a first experiment, *F*_0_ discrimination was investigated using complex tones that differed in harmonic content and phase configuration (sine, positive, or negative Schroeder). Musical training was associated with significantly better *F*_0_ discrimination of complex tones containing low-numbered harmonics for all groups of listeners. Part of this effect was caused by the fact that musicians were more robust than non-musicians to harmonic roving. Despite the benefit relative to their non-musicians counterparts, the older musicians, with or without HI, performed worse than the young musicians. In a second experiment, binaural sensitivity to temporal fine structure (TFS) cues was assessed for the same listeners by estimating the highest frequency at which an interaural phase difference was perceived. Performance was better for musicians for all groups of listeners and the use of TFS cues was degraded for the two older groups of listeners. These findings suggest that musical training is associated with an enhancement of both TFS cues encoding and *F*_0_ discrimination in young and older listeners with or without HI, although the musicians’ benefit decreased with increasing hearing loss. Additionally, models of the auditory periphery and midbrain were used to examine the effect of HI on *F*_0_ encoding. The model predictions reflected the worsening in *F*_0_ discrimination with increasing HI and accounted for up to 80 % of the variance in the data.

## **INTRODUCTION**

The effects of musical training on fundamental frequency (*F*_0_) discrimination have been largely investigated for young listeners with normal hearing (NH). Behavioral studies have shown that young NH musicians are two to six times more sensitive than non-musicians in complex-tone *F*_0_ discrimination (e.g., Micheyl et al. 2006; Bianchi et al. 2016a). Neuroimaging and electrophysiological studies have reported training-dependent plasticity in NH musicians at both cortical (Pantev et al. 1998; Schneider et al. 2002; Hyde et al. 2009; Foster and Zatorre 2010; Bianchi et al. 2017b) and subcortical stages (Musacchia et al. 2007; Wong et al. 2007; Parbery-Clark et al. 2009) of the auditory system. However, little is known about the effects of musical training for older listeners with or without hearing impairment. There is some evidence suggesting that musical training in the aging population leads to improved speech perception in noise and greater auditory working memory capacity (Parbery-Clark et al. 2011), as well as increased subcortical temporal precision (Parbery-Clark et al. 2012; Parbery-Clark et al. 2013). However, the observed effects of musical training on speech-in-noise performance are small (around 1 dB in speech reception threshold; Parbery-Clark et al. 2009, 2011) and controversial (Ruggles et al. 2014; Boebinger et al. 2015; Deroche et al. 2017; Madsen et al. 2017). This study focused on the effects of musical training both on *F*_0_ discrimination, for which the musicians’ benefit in young NH listeners is well established, and on binaural sensitivity to temporal fine structure (TFS) cues, estimated via the sensitivity to interaural phase differences (IPDs). The aim was to assess whether older and hearing-impaired (HI) listeners show a benefit of musical training comparable to that for young NH listeners and to clarify the extent to which the degradation in the encoding of peripheral cues (i.e., frequency selectivity and TFS) is a factor limiting musicians’ performance.

Possibly due to reduced frequency selectivity and/or degraded TFS processing, older and HI listeners with sensorineural hearing loss (SNHL) show a reduced ability to discriminate the *F*_0_ of complex tones with resolved harmonics (i.e., the overtones up to approximately the 8th; Plomp 1964) relative to young NH listeners (Moore and Peters 1992; Bernstein and Oxenham 2006b; Moore and Glasberg 2011). However, the ability of older and HI listeners to process high-numbered unresolved harmonics is not altered relative to NH listeners (Bernstein and Oxenham 2006b; Bianchi et al. 2016b). In fact, temporal envelope cues, which are typically seen as the main contributors to *F*_0_ encoding of unresolved harmonics (Oxenham et al. 2009), may be relatively more robust for HI listeners due to the reduced cochlear compression and the presence of more harmonic interactions on the basilar membrane (Kale and Heinz 2010; Henry et al. 2014; Bianchi et al. 2016b). As a consequence, the relative importance of spectral vs. temporal envelope cues may be altered in listeners with SNHL (Arehart 1994; Bianchi et al. 2016b). Since stronger cortical plasticity was observed in young NH musicians for complex tones with resolved harmonics than for tones with unresolved harmonics (Bianchi et al. 2017b), this study attempted to clarify whether musical training could help reestablish the relative importance of spectral and temporal envelope cues for older and HI listeners.

Two experiments were performed using three groups of listeners, young NH (YNH), older near-NH (ONH), and older HI (OHI), each including musicians and non-musicians. These groups were chosen based on the assumption that both ONH and OHI listeners would have degraded TFS processing (Moore et al. 2006b; Ross et al. 2007a), while the OHI listeners would also have degraded frequency selectivity but more robust coding of envelope cues relative to YNH. This design allowed observation of how the effect of musical training varied with an assumed degradation in the encoding of different pitch cues. In the first experiment, *F*_0_-discrimination performance was investigated using complex tones that differed in harmonic content to clarify how the effect of musical training varied when frequency selectivity and/or TFS sensitivity was reduced. In the second experiment, the ability to use TFS cues was assessed using an IPD detection task (Ross et al. 2007b), to clarify how age, hearing loss, and musical training affect TFS sensitivity. Additionally, phenomenological models of the auditory periphery (Zilany et al. 2009, 2014) and midbrain (Mao et al. 2013) were used to predict *F*_0_ discrimination based on neural representations, including average discharge rates and temporal patterns. The model allowed examination of the effects of hearing loss and harmonic phase on the complex-tone representations at the level of the auditory nerve (AN) and inferior colliculus (IC). Using psychophysical and modeling results, this study extends the findings of Bianchi et al. (2017a) and clarifies whether the degradation in the encoding of pitch cues may be counteracted by means of musical training.

## **METHODS**

### Listeners

**Fig. 1:**
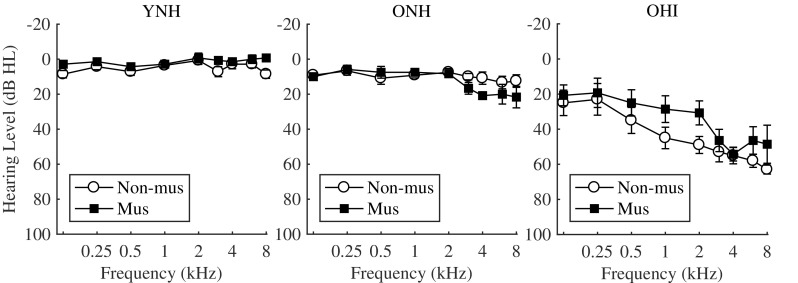
Mean hearing thresholds (± standard error) for the musicians and non-musicians within each group of listeners (young listeners with normal hearing: YNH; older listeners with near-normal hearing: ONH; older listeners with hearing impairment: OHI)

Fourteen YNH listeners (7 musicians, 7 non-musicians; mean age 25 ± 4 years), 12 ONH listeners (6 musicians, 6 non-musicians; mean age 61 ± 5 years), and 12 OHI listeners (7 musicians, 5 non-musicians; mean age 68 ± 6 years) participated in this study. The lower age limit of the ONH and OHI listeners was 55 years. Within each group of listeners, the mean age of musicians and non-musicians was not significantly different (unpaired *t* test; YNH, *p* = 0.294; ONH, *p* = 0.075; OHI, *p* = 0.35). All YNH listeners had hearing thresholds below or equal to 20 dB hearing level (HL) between 125 Hz and 8 kHz. The ONH listeners had hearing thresholds below or equal to 25 dB HL between 125 Hz and 4 kHz. The OHI listeners had hearing thresholds below or equal to 70 dB HL between 125 Hz and 4 kHz and pure-tone average (PTA) greater than 20 dB HL between 500 Hz and 8 kHz. Figure 1 depicts the mean hearing thresholds of musicians and non-musicians for the YNH, ONH, and OHI groups. A mixed-model ANOVA with frequency, group, and musicianship as fixed factors and subject as a random factor was fitted to the hearing thresholds of the tested ear. The effect of musicianship was not significant (*p* = 0.226), and neither were any of its interactions, i.e., the hearing thresholds of musicians and non-musicians were not significantly different. The interaction between group and frequency was significant (*p* < 0.0001). The hearing thresholds were significantly different at all audiometric frequencies between YNH and OHI, and between ONH and OHI listeners (*p* < 0.05 with Tukey’s method for *p* value adjustment), while they were not significantly different between YNH and ONH at frequencies lower than 4 kHz (*p* > 0.05). Musicians had at least 8 years of formal music education, had started musical training at or before the age of 12, and were actively playing music for more than 2 h per week. Non-musicians had less than 3 years of musical education and had stopped any type of musical training at least 3 years before participating in this study. One ONH listener underwent musical training for 6 years but stopped 40 years before his participation in this study and was recruited as a non-musician. His performance was within two standard deviations of the mean non-musicians’ performance.

### Experiment I: *F*_0_ Discrimination

A three-alternative forced choice (3-AFC) paradigm was used in combination with a weighted up-down method to estimate 75 % correct performance (Kaernbach 1991). In each trial, two intervals contained a reference complex tone with a fixed *F*_0_ (125 Hz) and one interval contained the target complex tone with a higher *F*_0_. The task was to select the interval containing the tone with the highest pitch. The difference in *F*_0_ between the reference and the target, ∆*F*_0_, was initially set to 20 % and was decreased after each correct response and increased after each incorrect response. After each correct response, ∆*F*_0_ was decreased by a factor of 2.2 until the first reversal, 1.7 until the third reversal, and 1.2 for the following reversals. After each incorrect response, ∆*F*_0_ was increased by three times the corresponding step size to achieve 75 % correct (Kaernbach 1991). The threshold for each condition, obtained as the geometric mean of ∆*F*_0_ at the last six reversals, was measured four times. The first repetition was considered as training and the last three were averaged (geometric mean) to estimate the final *F*_0_-discrimination threshold (or *F*_0_-difference limen; F_0_DL). Feedback was provided to the listeners.

Five conditions were tested in a randomized order: a resolved condition (RES, harmonics 3–9), an intermediate condition (INT, harmonics 10–16), two unresolved conditions (UN1, harmonics 17–23; UN2, harmonics 17–36), and a broadband condition (ALL, harmonics 3–36). The complex tones in the RES, INT, and UN1 conditions contained a total of 7 harmonics. All harmonics had equal amplitude, which was required for the construction of Schroeder-phase complex tones (Schroeder 1970). To avoid spectral edges as a discrimination cue, the lowest harmonic number was roved on an interval-by-interval basis, such that the three complex tones within each trial had lowest harmonic numbers of *N* − 1, *N*, or *N* + 1 in a random order, where *N* was the lowest nominal harmonic number in each condition (Houtsma and Smurzynski 1990; Bernstein and Oxenham 2003).

All signals were 300-ms complex tones embedded in broadband threshold equalizing noise (TEN, Moore et al. 2000). The complex tones were created by summing harmonic components either in sine, Schroeder positive (Schr+), or Schroeder negative phase (Schr−; Schroeder 1970). The phase of the *n*th harmonic was adjusted according to a modification of Schroeder’s (1970) equation, as suggested by Lentz and Leek (2001):

$$ {\varphi}_n=C\ \pi\ n\ \left(n-1\right)/{\mathrm{N}}_{tot}, $$

where *C* is a scalar and *N*_tot_ is the total number of harmonics in the complex tone. The sine-phase condition was obtained for *C* = 0, the Schr+ for C = + 1, and the Schr− for C = − 1. The Schroeder-phase complex tones have equally flat temporal envelopes (“external waveform”), while the sine-phase complex tones have a much peakier temporal envelope. However, the phase dispersion along the basilar membrane may cause the “internal waveform” of the Schr+ complex tone to be highly modulated, and that of the Schr− complex tone to be much flatter (Kohlrausch and Sanders 1995 [Fig. 18]; Lentz and Leek 2001). These hypothesized alterations in the internal waveform peakiness are due to the interaction of the stimulus phase curvature with the basilar-membrane phase curvature at a specific location (Kohlrausch and Sanders 1995; Lentz and Leek 2001; Oxenham and Dau 2001).

For the NH listeners, the TEN level was set to 55 dB SPL per equivalent rectangular bandwidth (ERB_N_; Glasberg and Moore 1990). For the OHI listeners, the level of the TEN per ERB_N_ was set to the maximum audiometric threshold up to 4 kHz. In order to keep the sensation level (SL) of the complex tones approximately constant across listeners, pure-tone detection in the TEN background was assessed at 1, 2, 3, and 4 kHz. For each listener, the mean detection threshold was calculated across the four frequencies and the level of each component of the complex tone was set to 12.5 dB SL re the mean threshold (Bernstein and Oxenham 2006b; Bianchi et al. 2016b). When necessary, the level of each harmonic was additionally increased to lead to at least 10 dB SL at each frequency. This procedure was carried out to ensure that all harmonics were audible up to 4 kHz for all listeners. At frequencies below 1 kHz, the sensation levels were equal to or higher than 10 dB SL because the OHI listeners had a flat or sloping hearing loss. The sound stimuli were delivered monaurally through headphones (Sennheiser HDA 200) using a Fireface UCX soundcard (RME, Germany) with a 48-kHz sampling rate and 16-bit resolution.

### Experiment II: IPD Detection

To obtain an estimate of interaural phase sensitivity, the highest frequency at which an IPD of 180° could be detected (Ross et al. 2007b) was measured using a 2-AFC paradigm with a two-down one-up tracking rule (71 % correct performance; Levitt 1971). For each trial, the reference interval contained four diotic pure tones (“AAAA”, IPD = 0°), each 400 ms in duration (including 20 ms cosine rise/fall ramps) with a 100-ms inter-stimulus interval (Füllgrabe et al. 2017). The target interval contained two diotic and two dichotic tones (IPD = 180°), presented in an interleaved manner (“ABAB”). The interval between reference and target was 333 ms. The listeners were instructed to select the interval containing the tones that were perceived as shifting in location inside the head, or the interval containing the tones that were simply perceived as different. The starting frequency was 500 Hz. The frequency was varied by a factor of 1.56 until the first upper reversal, 1.25 until the second upper reversal, and 1.1 for the following reversals. The final threshold was calculated as the mean frequency at the last six reversals. The lowest allowed frequency was 125 Hz. If the tracking variable reached a lower value than the minimum frequency three times, the run was interrupted and no threshold was measured (not a number, NaN).

The tones were presented at about 29 dB SL relative to the measured audiometric threshold (the sensation level was estimated based on conversions from dB HL to dB SPL according to standards ISO 389-7 and ISO 389-8). The levels were adjusted for each ear separately and the levels corresponding to frequencies in between two audiometric frequencies were obtained via linear interpolation. The experiment was carried out three times, and the final threshold was the mean of the three thresholds. Prior to carrying out the IPD experiment, the listeners had a short familiarization session (2 min) with a similar task, in which an interaural level difference (ILD) was introduced in the dichotic conditions instead of an IPD. All listeners could perform the ILD detection task, ensuring the understanding of the instructions.

A computer simulation was run to estimate chance performance in the IPD test. The 2-AFC procedure was simulated for a total of 3000 runs. For each stimulus presentation, the target interval was randomly selected. When the tracking variable reached lower values than 125 Hz three times, the run was interrupted and the threshold was set to NaN (as during the experiment). The final threshold was calculated as the mean threshold of three consecutive runs. In 47.2 % of the cases, there were three consecutive NaNs; in 41.4 % of the cases, there were two NaNs out of three runs; and in 10.2 % of the cases, there was a single NaN out of three runs. In only 1.2 % of the cases, there were no NaNs out of three consecutive runs. Hence, it was very likely (88.6 % of the cases) to have either two or three NaNs out of three runs when performance was at chance. The upper limit of the distribution of the final simulated thresholds was 400 Hz (5 % confidence level). This value was, thus, considered as the chance performance level of the mean of three runs in the IPD test.

### Model Predictions of *F*_0_ Discrimination

The reference and target complex tones, embedded in TEN and with the same *F*_0_, duration, and harmonic ranges as in experiment I, were used as inputs to a phenomenological model of the AN (Zilany et al. 2014). In each simulation, 96 two-interval trials (half with the target in the first interval, and half with the target in the second interval) were run for each condition to estimate the *F*_0_-discrimination threshold as in a 2-AFC procedure. Forty AN fibers (high spontaneous rate), logarithmically spaced from 125 Hz to 10 kHz, were included in the model. Only high spontaneous rate fibers were included in the model, since they constitute the majority of AN fibers, and they provide the major projections to the ascending pathway (Carney 2018). All conditions of experiment I were simulated (ALL, RES, INT, UN1, UN2) for the three harmonic phase configurations (Sine, Schr+, and Schr−). The model was run for *F*_0_ differences between reference and target, ∆*F*_0_, increasing from 0 to 24 % of *F*_0_ in steps of 2 %. The lowest harmonic number of the reference and target was roved, similar to experiment I. For each condition, six combinations of target and reference lowest harmonic number were used: (*N* + 1, *N*), (*N* + 1, *N* − 1), (*N*, *N* − 1), (*N*, *N* + 1), (*N* − 1, *N*), and (*N* − 1, *N* + 1). Each combination was repeated 16 times, resulting in 96 total trials per condition.

A bandpass modulation filter centered at 100 Hz with bandwidth of 100 Hz (*Q* = 1) was applied to the AN synapse output waveform as a simplified model of typical bandpass modulation tuning in the IC (Mao et al. 2013). This best modulation frequency was selected as it was near the stimulus *F*_0_ and in the middle of the distribution of best modulation frequencies in the IC (e.g., Krishna and Semple 2000). The IC model responses had rates that were proportional to the amplitude of the low-frequency fluctuations (near *F*_0_) of their inputs. Thus, the model effectively converted peripheral temporal information that was phase-locked to *F*_0_ into a rate profile across the IC model population.

A decision variable based on the rate differences combined across channels was used for both AN and IC population model responses to calculate a psychometric function of correct target identification as a function of ∆*F*_0_. Internal noise in the model was associated with spontaneous activity of the model’s high spontaneous rate fibers, which varied from trial to trial and over time within each trial (Zilany et al. 2009). In each trial, the target was correctly identified when the distance between the target rate in that trial and the reference mean rate across trials was larger than the distance between the reference rate in that trial and the reference mean rate across trials. The distance was based on a *d*′-like metric, as follows: for each frequency channel, the difference between the test interval response and the mean reference was normalized by the standard deviation of the reference responses. The channels were assumed to be independent, and an overall *d*′ was computed based on an optimal combination across channels, the square-root of the sum of (*d*′)^2^ for each frequency channel. That is, on each trial, the interval that elicited a population response that was most different from the mean reference response was selected as the target. The percentage correct for each ∆*F*_0_ was obtained as the number of correct trials over 96 total trials. The ∆*F*_0_ corresponding to 75 % correct performance (as in experiment I) on the fitted psychometric function was selected as the final simulated threshold.

Two simulations were run, one to estimate the *F*_0_-discrimination thresholds of NH listeners and one to estimate the thresholds of HI listeners. The stimulus levels used in the simulation were the mean levels used in experiment I for YNH and OHI listeners (YNH: TEN at 55 dB SPL/ERB_N_ and harmonics at 65 dB SPL; OHI: TEN at 59 dB SPL/ERB_N_ and harmonics at 77 dB SPL). Reduced sensitivity of inner hair cells (IHCs) and reduced cochlear amplification associated with outer hair cells (OHCs) in the HI simulation were selected to produce a threshold shift in the model that corresponded to the mean audiometric losses of the OHI listeners (Zilany and Bruce 2007). The threshold shift due to OHC impairment was adjusted to account for 2/3 of the entire threshold shift at each frequency (Jepsen and Dau 2011). In the NH simulation, no degradation of IHCs and OHCs was used.

### Statistical Analysis

Linear mixed models were used to analyze the effects of condition (i.e., harmonic numbers present in the stimulus), group, musicianship, and phase in experiment I and of group and musicianship in experiment II. In both models, subject was a random factor. The statistical analysis was computed using the statistical software R. All correlations were computed in Matlab.

## **RESULTS**

### Experiment I: *F*_0_ Discrimination

**Fig. 2:**
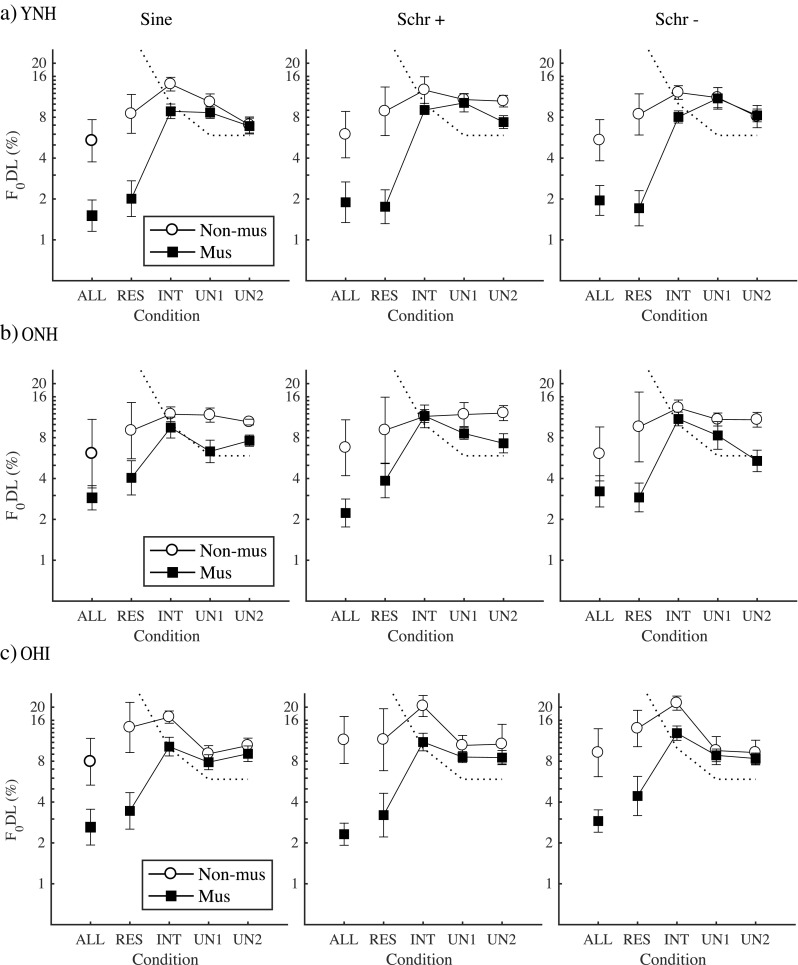
Mean *F*_0_-discrimination thresholds (F_0_DLs) for the three groups of listeners (**a** YNH; **b** ONH; **c** OHI), for musicians (filled symbols) and non-musicians (open symbols). Left panels: sine-phase configuration; middle panels: Schroeder +; right panels: Schroeder −. Error bars depict the standard error of the mean. The dashed line depicts the predicted thresholds (66.7 % correct) based on spectral edge as a discrimination cue, rather than *F*_0_ cues.

The mean F_0_DLs for the three groups of listeners are presented in Fig. 2 (Fig. 2a: YNH; Fig. 2b: ONH; Fig. 2c: OHI listeners), for musicians (filled symbols) and non-musicians (open symbols). Thresholds were lowest for the ALL and RES conditions and increased for the INT and UN conditions, consistent with earlier findings of a more salient pitch percept for tones with low-numbered harmonics (e.g., Houtsma and Smurzynski 1990; Bernstein and Oxenham 2003, 2006a, b; Bianchi et al. 2016b). This effect was more pronounced for the musicians, whose thresholds in the RES and UN1 conditions were, on average, 1.8 % and 9.9 % for YNH, 3.6 % and 7.7 % for ONH, and 3.7 % and 8.4 % for OHI, respectively. For the non-musicians, the thresholds in the RES and UN1 condition were, on average, 8.6 % and 10.8 % for YNH, 9.2 % and 11.5 % for ONH, and 13.1 % and 9.7 % for OHI, respectively.

A mixed-model ANOVA, with condition, musicianship, group, repetition, and phase as fixed factors and subject as random factor, was fitted to the log-transformed F_0_DLs. Repetition and phase were not significant (repetition [*F*(2,1541) = 0.38; *p* = 0.683]; phase [*F*(2, 1532) = 1.30; *p* = 0.272]), neither were any of their interactions, so these factors were removed from the final model. The reduced model confirmed a significant effect of condition [*F*(4, 1537) = 174.67; *p* < 0.0001] and musicianship [*F*(1, 32) = 18.67; *p* = 0.0001], as well as a significant interaction between condition and musicianship [*F*(4, 1537) = 51.16; *p* < 0.0001]. The interaction between musicianship and group was not significant [*F*(2,32) = 0.09; *p* = 0.918], suggesting a similar effect of musicianship across groups. While the main effect of group was not significant [*F*(2, 32) = 1.07; *p* = 0.356], there was a significant interaction between group and condition [*F*(8, 1537) = 5.29; *p* < 0.0001]. The interaction between musicianship, group, and condition was also significant [*F*(8, 1537) = 4.85; *p* < 0.0001].

Post hoc tests, using Tukey’s method for *p* value adjustments, showed a significant effect of musicianship for the ALL and RES conditions, for YNH (ALL: *p* < 0.0001; RES: *p* < 0.0001), ONH (ALL: *p* = 0.004; RES: *p* = 0.002), and OHI listeners (ALL: *p* = 0.0001; RES: *p* = 0.0001). A significant effect of musicianship also occurred for the INT condition for OHI listeners (*p* = 0.041), while it was not significant for the other two groups (YNH: *p* = 0.126; ONH = 0.698). No significant effect of musical training occurred for the UN conditions, in contrast to previous studies with YNH listeners and sine-phase complex tones (Bianchi et al. 2016a, 2017b). The effect of group was significant for the RES condition between musicians YNH and ONH (*p* = 0.024) and between musicians YNH and OHI (*p* = 0.020). No significant group differences were observed for the other conditions, nor between ONH and OHI listeners. The effect of condition was significant for YNH musicians between RES and INT (*p* < 0.0001), RES and UN1 (*p* < 0.0001), RES and UN2 (*p* < 0.0001), but not between ALL and RES (*p* = 0.999). For YNH non-musicians, the thresholds for the RES condition were only significantly different from those for the INT (*p* = 0.011) and ALL (*p* = 0.002) conditions. The significant decrease in the ALL relative to the RES condition suggests a less distracting effect of harmonic roving with increasing the total number of harmonics (Moore et al. 2006a). Also for the ONH and OHI musicians, the thresholds for the RES condition were significantly different from those for the INT (*p* < 0.0001), UN1 (*p* < 0.0001), and UN2 (*p* < 0.0001) conditions. The difference between RES and ALL thresholds was significant for OHI musicians (*p* = 0.008), but not for ONH musicians (*p* = 0.058). For ONH non-musicians, the RES thresholds were significantly different only from the ALL thresholds (*p* = 0.0081). For OHI non-musicians, the RES thresholds were significantly different only from the INT thresholds (*p* = 0.006).

The dashed line in Fig. 2 shows the predicted thresholds (66.7 % correct) if performance had solely been based on spectral edge cues rather than *F*_0_ cues. Since the harmonic components used in experiment I had equal amplitude and the conditions were defined in terms of a fixed range of harmonic numbers (and not a fixed frequency range), spectral edge cues may have helped in the discrimination task. Despite the harmonic roving, this could have occurred in two cases out of three: when the lowest harmonic number of the target was N + 1 (1/3 cases: 33 %), spectral edge cues always helped in the discrimination; when the lowest harmonic number of the target was *N* (1/3 cases: 33 %), spectral edge cues helped in the discrimination task only when ∆*F*_0_/*F*_0_ > 1/*N* (Bernstein and Oxenham 2003). Hence, if the listeners had solely used the frequency of the lowest harmonic as a discrimination cue, they would have achieved 66.7 % correct performance when ∆*F*_0_/*F*_0_ > 1/*N* (i.e., above the dashed line in Fig. 2). Although 66.7 % is lower than the tracked 75 % correct performance, it is possible that thresholds markedly above the dashed line were based on spectral edge cues, rather than *F*_0_ cues (Bernstein and Oxenham 2003). Since most thresholds in the UN conditions were above the dashed line, it cannot be excluded that for these conditions, the listeners used spectral edges as a cue.

### Experiment II: IPD Detection

**Fig. 3:**
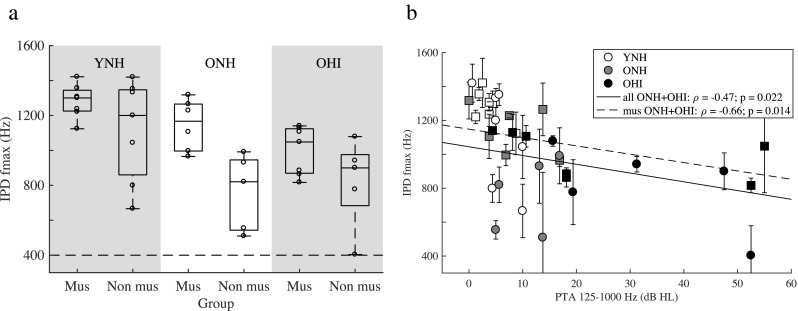
**a** Highest frequency (fmax) at which an interaural phase difference (IPD) of 180° can be detected for YNH, ONH, and OHI listeners (experiment II). The dashed line indicates the chance performance level. **b** Scatter plot and Spearman correlation between the IPD fmax and the PTA of the older listeners (solid regression line: ONH and OHI non-musicians; dashed regression line: ONH and OHI musicians). The data of YNH listeners are shown for comparison purpose, but were not included in the correlation. Data for musicians are depicted by square symbols, for non-musicians by circles (open symbols: YNH; gray-filled symbols: ONH; black-filled symbols: OHI)

Figure 3a depicts the highest frequency (fmax) at which an IPD was detected for each listener group. YNH musicians were sensitive to the IPD shift, on average, up to 1281 Hz, while YNH non-musicians were sensitive up to 1116 Hz. These values are similar to the thresholds previously obtained for YNH listeners (Ross et al. 2007a, b; Füllgrabe et al. 2017; Füllgrabe and Moore 2017). The highest frequency for sensitivity to IPD cues decreased for the ONH listeners (musicians 1146 Hz; non-musicians 761 Hz), and for the OHI listeners (musicians 999 Hz; non-musicians 820 Hz). A mixed-model ANOVA, with repetition, group, and musicianship as fixed factors and subject as random factor, was fitted to the data. Repetition was not significant (*F*(2,70) = 0.11; *p* = 0.897), nor were any of its interactions, and it was removed from the model. The reduced model showed a significant effect of both fixed factors (group: *F*(2, 33) = 7.89, *p* = 0.002; musicianship: *F*(1, 33) = 12.42, *p* = 0.001). The interaction was not significant (*F*(2,31) = 1.07; *p* = 0.357).

Post hoc tests, using Tukey’s method for *p* value adjustments, showed a significant effect of group between YNH and ONH (*p* = 0.014), YNH and OHI (*p* = 0.003), but not between ONH and OHI listeners (*p* = 0.849), suggesting that age decreased the sensitivity to TFS cues (Ross et al. 2007a; Füllgrabe et al. 2017). Although the thresholds of ONH and OHI listeners were not significantly different, a low but significant correlation was found between the combined thresholds of ONH and OHI listeners and their PTA between 125 Hz and 1 kHz (Spearman correlation: *ρ* = − 0.47, *p* = 0.022, solid line in Fig. 3b), in agreement with the findings of Füllgrabe and Moore (2017). The correlation was also present when only considering the ONH and OHI musicians (*ρ* = − 0.66, *p* = 0.014, dashed line in Fig. 3b). No correlation was found when only considering the ONH and OHI non-musicians.

The dashed horizontal line in Fig. 3a depicts the simulated chance performance level (400 Hz). All listeners except one performed the test above chance level and only the threshold of one OHI listener (non-musician) was close to chance performance (403 Hz). One ONH non-musician could not perform the task and thus no threshold is reported. One ONH non-musician had a skipped measurement (i.e., the tracking variable reached lower values than the minimum frequency three times). Hence, only the mean of two repetitions was reported for this listener.

### IPD Detection and *F*_0_ Discrimination

**Fig. 4:**
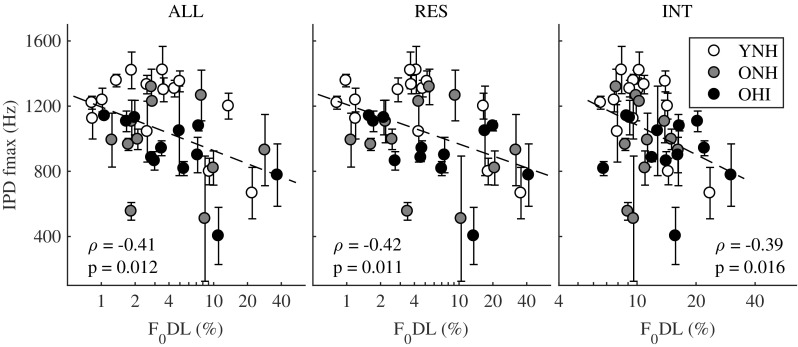
Scatter plot and Spearman correlation between IPD fmax values (experiment II) and the F_0_DLs (experiment I), for the ALL (left panel), RES (middle panel), and INT (right panel) conditions. For readability, the *x*-axis of the right panel spans a restricted range of F_0_DLs

Figure 4 shows the scatter plots and Spearman correlations between the IPD fmax thresholds and the F_0_DLs from experiment I, averaged across phase conditions, for the ALL (left panel), RES (middle panel), and INT (right panel) conditions. After Bonferroni correction with *n* = 3 comparisons (significance for *p* < 0.0167), significant correlations were found for the ALL (*ρ* = − 0.41; *p* = 0.012), RES (*ρ* = − 0.42; *p* = 0.011), and INT (*ρ* = − 0.39; *p* = 0.016) conditions. The three correlation coefficients were not significantly different (Fisher’s *r*-to-*z* transformation, *p* > 0.05). There was no significant correlation for the UN conditions. This finding suggests that TFS cues may play a role for *F*_0_ discrimination of complex tones containing low and intermediate numbered harmonics (Moore and Moore 2003; Santurette and Dau 2011).

### Model Predictions of *F*_0_ Discrimination

**Fig. 5:**
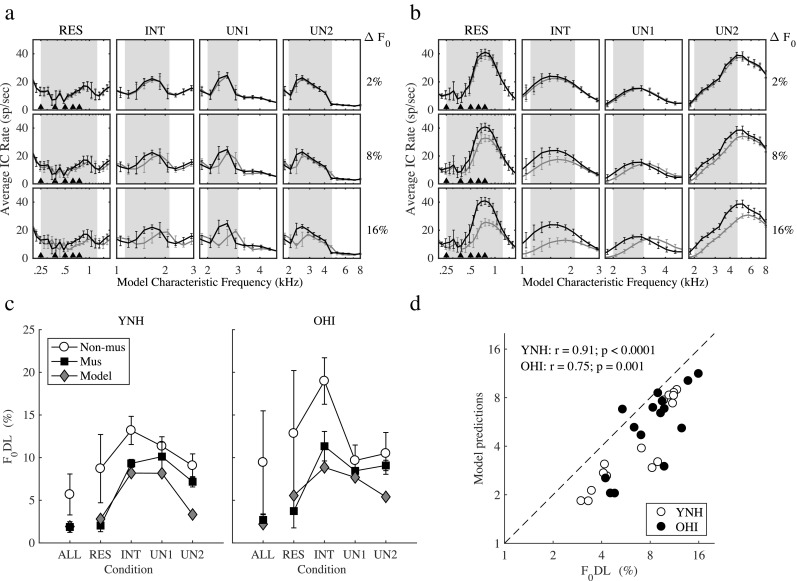
**a**, **b** Average IC rates for the reference (black curve) and target (gray curve) complex tones (sine-phase condition), for three ΔF_0_s (top panels: 2 %; middle panels: 8 %; bottom panels: 16 %), and four harmonic ranges (RES, INT, UN1, and UN2). In each panel, the frequency region of the complex tone (from *N* − 1 to *N* + *N*_tot_) is highlight in gray. The triangular symbols in the RES condition depict the harmonics from the 2^nd^ to the 6^th^. **a** Simulation for NH listeners. **b** Simulation for HI listeners. **c** Measured (open symbols: non-musicians; filled symbols: musicians) vs. predicted (gray-filled symbols) F_0_DLs, for YNH (left panel) and OHI (right panel) listeners, averaged across the three phase configurations. **d** Scatter plot and Pearson correlation between the model predictions and the F_0_DLs measured in experiment I, for all the 15 conditions (3 phase conditions, 5 harmonic ranges)

The Zilany et al. (2014) and Mao et al. (2013) models of the AN and IC, respectively, were used to clarify the effect of hearing loss on neural representations of complex tones. Panels a and b in Fig. 5 show the average discharge rate at the IC level (i.e., after applying the bandpass modulation filter at 100 Hz) in response to sine-phase complex tones, simulated for NH (Fig. 5a) and HI (Fig. 5b) listeners, for the RES, INT, UN1, and UN2 conditions. The black and gray lines represent the responses to the reference and the target stimuli, respectively, with ∆*F*_0_ increasing from 2 % (top panels) to 16 % (bottom panels). In the NH simulation, clear peaks and dips in the spiking rate occurred up to the 4^th^ harmonic number for the RES condition (the black triangles indicate harmonic numbers from 2 to 6). Interestingly, the modulation cues were strongest (i.e., peaks in the model IC average rate) for frequency channels tuned between resolved harmonics, in agreement with previous findings (Henry et al. 2016). This pattern is explained by reduced *F*_0_-related fluctuations in NH AN responses near the harmonics, due to synchrony capture (Deng et al. 1987; Zilany and Bruce 2007). In the HI simulation, only the first 2–3 harmonics elicited peaks and dips in the spiking rate, consistent with reduced synchrony capture (Miller et al. 1997); above that frequency, the harmonics interacted on the basilar membrane, giving rise to a smooth pattern. The average rates in the HI simulation were elevated over a broader range of characteristic frequencies than in the NH simulation for the INT, UN1, and UN2 conditions (Fig. 5b), due to the reduced frequency selectivity, reduced cochlear compression, and higher stimulus levels as compared to the NH simulation. These model responses are consistent with enhanced AN phase locking to amplitude-modulated stimuli in ears with SNHL (Henry et al. 2014), as well as with psychoacoustical findings of enhanced envelope coding in HI listeners (Moore et al. 1996; Bianchi et al. 2016b).

*F*_0_-discrimination thresholds, i.e., the ∆*F*_0_ corresponding to 75 % correct performance, were predicted based on the model IC rate differences between the reference and target. The model predictions for NH and HI listeners, averaged across the three phase configurations, are presented in Fig. 5c (gray diamonds), together with the F_0_DLs obtained in experiment I for the musicians (black squares) and non-musicians (open circles). The predictions were averaged across the three phase configurations since there was no effect of phase on the predicted F_0_DLs. The model predicted the musicians’ performance quite accurately in the ALL, RES, INT, and UN1 conditions for both YNH and OHI listeners, while it predicted lower thresholds in the UN2 condition. The mean absolute error (MAE) between the model predictions and the mean F_0_DLs of musicians and non-musicians was 2.3 % of *F*_0_ (i.e., 2.9 Hz) for the NH simulation and 2.9 % of *F*_0_ (i.e., 3.6 Hz) for the HI simulation (when considering all 15 data points: three phase configurations and five harmonic ranges). For the musicians’, the MAE was only 1.6 % of *F*_0_ for the NH and 1.8 % of *F*_0_ for the HI simulation. Figure 5d shows a scatter plot of model predictions (all 15 data points) and the data (filled symbols: mean of all YNH listeners; open symbols: mean of all OHI listeners). The Pearson’s correlation between the predictions and the data was *r* = 0.9 for YNH, and *r* = 0.76 for OHI listeners. The correlation coefficient for OHI listeners was lower than for YNH because the model underestimated the thresholds of the OHI listeners in the sine-phase configuration for the INT and UN2 conditions. It should be mentioned that *F*_0_-discrimination thresholds could not be predicted solely based on the average rates of the model AN responses. However, AN responses carried the temporal information related to *F*_0_ fluctuations that allowed the average rates of the IC model to predict the discrimination thresholds.

### Effect of Hearing Loss on *F*_0_ Discrimination: Behavioral Data and Model Predictions

**Fig. 6:**
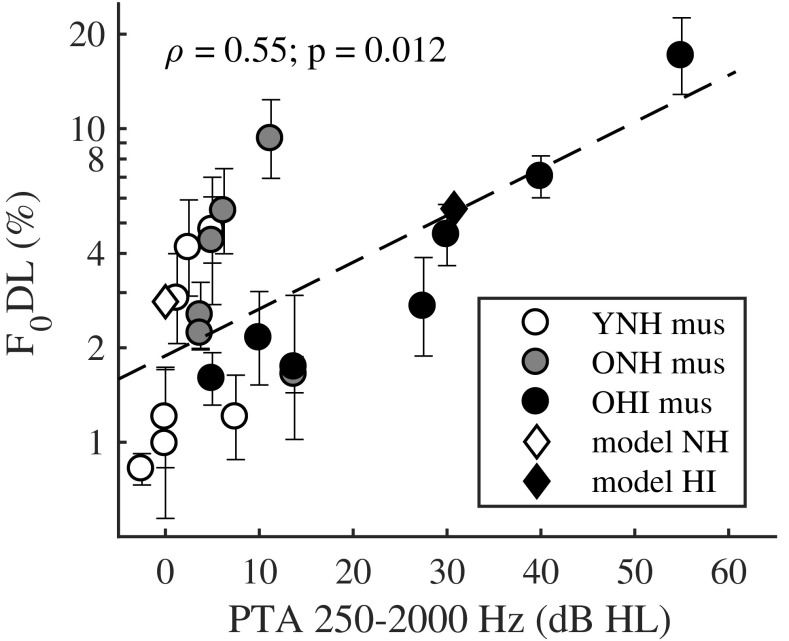
Scatter plot and Spearman correlation between the F_0_DLs for the RES condition, averaged across phase configurations, and the PTA between 250 Hz and 2 kHz of all musicians (open circles: YNH; gray-filled circles: ONH; black circles: OHI). The model predictions for the RES condition, averaged across phase configurations, for NH (PTA = 0 dB HL) and HI listeners (PTA = 31 dB HL), are depicted by the open diamond and filled diamond, respectively

The individual performance of all musicians in the RES condition, averaged across phase configurations, is shown in Fig. 6 as a function of the PTA between 250 Hz and 2 kHz (i.e., about the frequency range of the RES complex tones). The performance of musicians worsened with increasing hearing loss (Spearman correlation for all musicians: *ρ* = 0.55; *p* = 0.012; Spearman correlation for OHI musicians: *ρ* = 0.96; *p* = 0.003). The correlation remained significant even when removing the OHI listener with the greatest hearing loss (all musicians: *ρ* = 0.48; *p* = 0.039; OHI musicians: *ρ* = 0.94; *p* = 0.017). The model predictions for NH and HI, averaged across phase configurations, are also presented in Fig. 6 (NH: open diamond; HI: filled diamond). The model could account for the difference in performance between an average NH listener with a PTA of 0 dB HL (predicted F_0_DL = 2.8 %) and a listener with a mild hearing loss up to 2 kHz (PTA = 31 dB HL; predicted F_0_DL = 5.6 %).

## **DISCUSSION**

### Effect of Musical Training on *F*_0_ Discrimination

The musicians’ *F*_0_-discrimination performance obtained in experiment I was better than that of non-musicians for all three groups of listeners. The benefit of musicianship was significant for the ALL and RES conditions (i.e., for complex tones containing low-numbered harmonics), and for the INT condition for OHI listeners. In previous studies, a significant benefit of musicianship was also observed for YNH listeners for unresolved complex tones (Bianchi et al. 2016a; Bianchi et al. 2017b). In those studies, spectral edge cues were minimized by filtering the complex tones in a fixed frequency region such that the spectral centroid was the same for the reference and target tones, thus avoiding the need for harmonic roving. In the current study, the complex tones consisted of equal-amplitude harmonics, with a fixed range of harmonic numbers, yielding strong spectral edge cues. This was a necessity of the study design, to allow investigation of *F*_0_ discrimination with Schroeder-phase complexes. To avoid discrimination based on spectral edge cues rather than *F*_0_ cues, the lowest harmonic number was roved by ± 1 across intervals. However, for high harmonic numbers (UN condition), harmonic roving could not completely prevent the listeners from using spectral edges as a discrimination cue (Moore et al. 2006a; Bernstein and Oxenham 2003). The availability of spectral edges as a cue may explain the absence of differences in performance between musicians and non-musicians for the UN conditions, in contrast to Bianchi et al. (2016a, 2017b). According to Seither-Preisler et al. (2007), when both *F*_0_ and spectral edge cues are available, musicians tend to use *F*_0_ cues rather than spectral edges. In contrast, non-musicians tend to use spectral edge cues. Thus, it might be that, while musicians used *F*_0_ cues also for the UN conditions, non-musicians used spectral edges as a cue and performed as well as the musicians. It is also possible that both musicians and non-musicians used spectral edge cues and performed similarly.

A second consequence of harmonic roving was that the thresholds of the non-musicians for the RES and ALL conditions were much higher (8.6 % and 5.6 %, respectively) than the F_0_DLs obtained in previous studies (about 1 to 2 %) for complex tones with resolved harmonics presented at similar sensation levels as in the current study (Bernstein and Oxenham 2006a; Oxenham et al. 2009; Bianchi et al. 2016a). In contrast, the thresholds of the musicians for the RES condition were only slightly higher (1.8 %) than the F_0_DLs obtained for musicians for similar stimulus conditions but without harmonic roving (about 1 %; Bianchi et al. 2016a, 2017b). Hence, the random changes in the lowest harmonic number may have been more distracting for the non-musicians than for the musicians. In favor of this hypothesis, the F_0_DLs were significantly lower (better) for the ALL than for the RES condition for the non-musicians (YNH and ONH), but not for the musicians (YNH and ONH). This may be due to the presence of more harmonics in the ALL condition, which reduced the distracting effect of harmonic roving for the non-musicians (Moore et al. 2006a). Overall, the musicians seemed to be more robust than the non-musicians to the effect of harmonic roving in the ALL and RES conditions, suggesting that the encoding of *F*_0_ for tones containing low-harmonic numbers may be less susceptible to changes in harmonic number—perhaps as a consequence of a stronger *F*_0_ encoding mechanism for low-numbered harmonics in musicians (Wong et al. 2007; Seither-Preisler et al. 2007; Bianchi et al. 2017b). The benefit of musicianship was more pronounced in the YNH group, but was also present in the ONH and OHI groups, suggesting that musical training could be associated with better *F*_0_ discrimination of low-numbered harmonics also for older listeners with or without hearing loss.

### Effect of Musical Training on TFS Processing

The outcomes of experiment II showed that the sensitivity to TFS cues decreased for the ONH and OHI listeners relative to the YNH group, in agreement with Ross et al. (2007a and b) and Füllgrabe and Moore (2017). The novel finding, here, was that the musicians in each group of listeners were able to detect the IPD change up to higher frequencies than the non-musicians. This key finding suggests that the effect of musical training could counteract the decrease in TFS sensitivity that would normally start before midlife (> 45 years old, Ross et al. 2007a). Although this is one of the first behavioral studies to show greater TFS sensitivity in musicians, a comparable effect of musical training was found in a previous electrophysiological study, in which greater neural synchrony to a speech syllable was observed in the brainstem of older musicians relative to non-musicians (Parbery-Clark et al. 2012). Hence, the increased performance of musicians observed in experiment II may be related to increased temporal synchrony at the brainstem level, which would increase the upper frequency limit for an IPD detection. However, the musicians’ advantage in IPD detection could also be related to higher-level cognitive factors, such as attention and auditory working memory, which have been shown to be enhanced in musicians (Zatorre et al. 1994; Parbery-Clark et al. 2011), as well as to a general greater listening ability.

It should be noted that musical training did not completely preserve sensitivity to TFS cues in the presence of SNHL (see correlation with PTA in Fig. 3b). Decreased sensitivity to IPDs in listeners with SNHL may be a consequence of decreased frequency selectivity, leading to alterations in the cochlear traveling wave and, thus, to changes in the phase difference across cochlear locations (Sayles and Heinz 2017). Broader cochlear tuning leads to more coincident responses across a wider range of cochlear locations near the target frequency (Carney 1994). These alterations in across-fiber spatiotemporal coding may be relevant for processing interaural time and phase differences (Shamma et al. 1989; Joris et al. 2006). Additionally, decreased sensitivity to TFS cues may be ascribed to IHC dysfunction (Buss et al. 2004; Sayles and Heinz 2017), reducing the accuracy in the encoding of the stimulus waveform at the AN. Finally, age-related changes along the auditory system may also affect the encoding of TFS cues (Frisina 2010). Although in the current study no significant interaction of musicianship and group was observed, possibly due to the relatively small sizes of the six groups, the effect of age seemed to be the main factor associated with the decreased performance of non-musicians, while hearing loss appeared to be the main factor associated with the decreased performance of musicians (Fig. 3a, b). Although it remains speculative, it is possible that musical training can compensate for deficits in the sensory encoding of TFS cues originating at subcortical or central stages of the auditory system (e.g., age-related deficits in neural synchrony; Frisina 2010; Zendel and Alain 2012) but not at cochlear stages (e.g., reduced frequency selectivity and IHC dysfunction).

### Effect of Hearing Loss and Age on *F*_0_ Discrimination and TFS Processing

The outcomes of experiment I showed a significant effect of group in the RES condition only for the musicians (YNH vs. ONH; YNH vs. HI). No group differences were observed for the non-musicians, in contrast to previous studies, where *F*_0_ discrimination was shown to worsen with age and hearing loss for complex tones with low-numbered harmonics (Moore and Peters 1992; Bernstein and Oxenham 2006b; Moore and Glasberg 2011; Bianchi et al. 2016b). The absence of group differences for the non-musicians might be ascribed to the random changes in the lowest harmonic number, which were more distracting for non-musicians than for musicians. The musicians, who were, instead, more robust to the effect of harmonic roving, showed a worsening in *F*_0_-discrimination performance with both age and hearing loss (Fig. 6). Similarly, the outcomes of experiment II showed group differences between YNH and ONH, as well as YNH and OHI. The sensitivity to TFS cues also decreased with increasing hearing loss for the older listeners (Fig. 3b), especially for the musicians, who seemed to be affected more by hearing loss than age.

The worsening in *F*_0_ discrimination predicted by the HI model (Fig. 6) can be mainly ascribed to two factors: reduced synchrony capture and increased phase locking to the *F*_0_ fluctuations. The former causes the dips in the average IC rate for channels tuned near the harmonics (Fig. 5b) to be shallower for the HI than for the NH responses. The reduction in the dips in the HI responses reduced the difference between the population responses across the two intervals of each trial and contributed to the worsening in performance in the HI model for the RES condition. Concerning the second factor, the HI model responses showed enhanced envelope coding in single AN fibers, in agreement with physiological findings (Kale and Heinz 2010; Henry et al. 2014). However, stronger *F*_0_ responses (Fig. 5b) reduce the difference in the responses across intervals, which explains the worsening in performance in the HI model.

### Temporal Fine Structure vs. Envelope Coding

A significant correlation was found between the IPD fmax values and the F_0_DLs for the ALL, RES, and INT conditions, suggesting that TFS cues may play a role in *F*_0_ discrimination of complex tones containing low and intermediate numbered harmonics (Moore and Moore 2003; Santurette and Dau 2011). However, the correlation was not strong and only about 17 % of the variance in the F_0_DLs could be explained by sensitivity to binaural TFS cues. Because both the IPD fmax values and the F_0_DLs were correlated with the low-frequency PTA, it may be that a decrease in frequency selectivity led to changes in across-fiber spatiotemporal coding, which impaired both TFS processing and *F*_0_ discrimination (Sayles and Heinz 2017). Hence, the correlation between IPD fmax values and the F_0_DLs may be driven by the broadening of the auditory filters, and/or it may indicate that indeed TFS cues play a role in the *F*_0_ discrimination of complex tones with low and intermediate harmonic numbers (Moore and Moore 2003; Santurette and Dau 2011). It is worth noticing that the model predicted ~ 80 % of the variance of the F_0_DLs based on AN phase locking to *F*_0_ fluctuations, and not TFS cues. However, since both TFS and F_0_-related information are available in AN responses (Kale et al. 2014), and could be used for *F*_0_ discrimination of complex tones with low-numbered harmonics (Moore and Moore 2003; Santurette and Dau 2011), this study cannot disentangle the relative contribution of TFS and envelope cues.

### Phase Effects in *F*_0_ Discrimination: Behavioral Data and Model Predictions

**Fig. 7:**
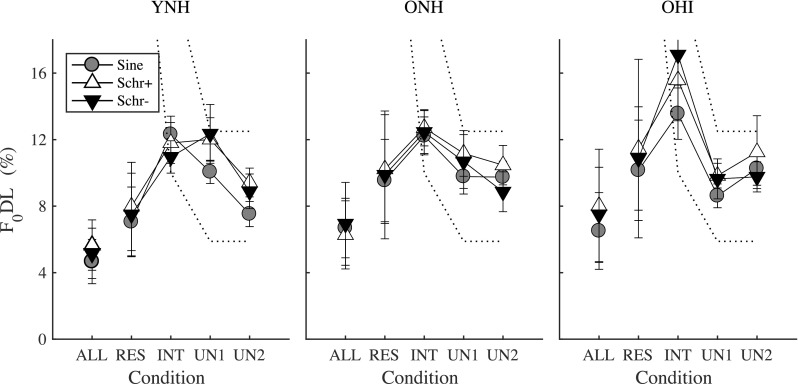
Effect of phase on *F*_0_ discrimination, for YNH (left panel), ONH, and OHI (right panel). For each group, the F_0_DLs depict the mean threshold for all listeners (musicians and non-musicians). The two dashed lines depict the predicted thresholds based on the frequency of the lowest harmonic number (*f*_N_) as a discrimination cue, rather than *F*_0_ cues. The lower dashed line depicts the Δ*F*_0_ for which the *f*_N_ of the target is equal to the *f*_N + 1_ of the reference (i.e., when Δ*F*_0_ = 1/N*100). Above this line, 66.7 % correct can be achieved using spectral edge cues. The higher dashed line depicts the Δ*F*_0_ for which the *f*_N − 1_ of the target is equal to the *f*_N + 1_ of the reference (i.e., when Δ*F*_0_ = 2/(*N* − 1)*100). Above this line, 100 % correct can be achieved using spectral edge cues. F_0_DLs between the two dashed lines may be based on spectral edge cues, rather than *F*_0_ cues

Experiment I did not show a significant difference in *F*_0_ discrimination between sine and Schroeder-phase complex tones, in contrast to Houtsma and Smurzynski (1990). Figure 7 depicts the F_0_DLs obtained in the three phase configurations, for the YNH (left panel), ONH (middle panel), and OHI (right panel) listeners. For the YNH listeners, the F_0_DLs for Schr− phase were higher than those for sine phase by 2.5 % of *F*_0_ (i.e., about 3 Hz) for the UN1 condition and 1.5 % of *F*_0_ (i.e., 1.8 Hz) for the UN2 condition. For both these conditions, the lowest harmonic number of the complex tone was 17. In the study of Houtsma and Smurzynski (1990), the difference in F_0_DLs between sine and Schr− was about 2 Hz (i.e., 1 % of the *F*_0_), when the lowest harmonic number was 16. Only four listeners, musically trained and with considerable experience in pitch experiments, participated in their study. Although the phase effects obtained in the current study with 14 YNH listeners (7 musicians, 7 non-musicians) were similar in size to those of Houtsma and Smurzynski (1990), they were not statistically significant. Additionally, no difference in F_0_DLs was obtained between the Schr+ and Schr− phase configurations, in contrast to the initial expectations based on the hypothesized alterations in the internal waveform peakiness (Kohlrausch and Sanders 1995; Lentz and Leek 2001). Some possible explanations for the absence of significant phase effects are discussed below.

First, a noise level high enough to mask distortion products was used in the current study, in combination with a low sensation level, which has been shown to lead to higher F_0_DLs (Oxenham et al. 2009). When the F_0_DLs are high, in this case for F_0_DLs between the two dashed lines (Fig. 7), spectral edge cues might help in the discrimination task for the INT and UN conditions. In previous studies, no significant phase effects were observed for either NH or HI listeners when the F_0_DLs were large and performance could have been based on spectral edge cues (Moore et al. 2006b; Bernstein and Oxenham 2003; Oxenham et al. 2009). Second, when the level of the background noise is not high enough to mask possible distortion products (e.g., Houtsma and Smurzynski 1990; Moore et al. 2006a), phase may have a larger effect on F_0_DLs, as discussed in Oxenham et al. (2009). This effect could be ascribed to stronger distortion products in one phase configuration than in the other (Pressnitzer and Patterson 2001). Hence, the phase effects observed by Houtsma and Smurzynski (1990) may have been enhanced by the presence of distortion products in the sine-phase, but not Schroeder phase, condition. The absence of significant phase effects both in the data and in the model predictions suggests that the interactions between the stimulus and the basilar-membrane phase curvature may be more complex to explain than previously thought (Kohlrausch and Sanders 1995; Lentz and Leek 2001; Oxenham and Dau 2001; Wojtczak and Oxenham 2009; Wojtczak et al. 2015).

## **CONCLUSIONS**

A benefit of musical training comparable to that for YNH listeners was obtained for ONH and OHI listeners, for both *F*_0_ discrimination and IPD detection. These results suggest that musical training was associated with greater sensitivity in the encoding of both *F*_0_ and TFS cues. Despite the enhancement relative to their non-musicians counterparts, the performance of the older musicians decreased with increasing hearing loss. The findings of this study suggest that music-training paradigms may be investigated as a tool for improving auditory perceptual skills, particularly for older listeners with mild to moderate hearing loss.
